# A Fiber Optic Ammonia Sensor Using a Universal pH Indicator

**DOI:** 10.3390/s140304060

**Published:** 2014-02-27

**Authors:** Adolfo J. Rodríguez, Carlos R. Zamarreño, Ignacio R. Matías, Francisco. J. Arregui, Rene F. Domínguez Cruz, Daniel. A. May-Arrioja

**Affiliations:** 1 Fiber and Integrated Optics Laboratory, Electronics Engineering Department, UAM Reynosa Rodhe, Universidad Autónoma de Tamaulipas, Carr. Reynosa-San Fernando S/N, Reynosa, Tamaulipas 88779, Mexico; E-Mails: rfdominguez@uat.edu.mx (R.F.D.C.); darrioja@uat.edu.mx (D.A.M.-A.); 2 Departamento de Ingeniería Eléctrica y Electrónica, Universidad Pública de Navarra, Edif. Los Tejos, Campus Arrosadia, Pamplona España 31006, Spain; E-Mails: carlos.ruiz@unavarra.es (C.R.Z.); natxo@unavarra.es (I.R.M.); parregui@unavarra.es (F.J.A.)

**Keywords:** fiber optics sensor, ammonia sensor, optical sensor

## Abstract

A universal pH indicator is used to fabricate a fiber optic ammonia sensor. The advantage of this pH indicator is that it exhibits sensitivity to ammonia over a broad wavelength range. This provides a differential response, with a valley around 500 nm and a peak around 650 nm, which allows us to perform ratiometric measurements. The ratiometric measurements provide not only an enhanced signal, but can also eliminate any external disturbance due to humidity or temperature fluctuations. In addition, the indicator is embedded in a hydrophobic and gas permeable polyurethane film named Tecoflex^®^. The film provides additional advantages to the sensor, such as operation in dry environments, efficient transport of the element to be measured to the sensitive area of the sensor, and prevent leakage or detachment of the indicator. The combination of the universal pH indicator and Tecoflex^®^ film provides a reliable and robust fiber optic ammonia sensor.

## 1. Introduction

Ammonia is a natural gas employed in the automotive and chemical industry and medical analysis [1]. Due to its potential hazard to human beings, even at small concentrations, real time environmental monitoring of ammonia is a critical issue in closed environments. Ammonia has a strong smell that can be perceived at concentrations close to 50 ppm and which induces irritation in the upper respiratory tract and chronic cough [2]. On the other hand, prolonged exposure to ammonia concentrations below 25 ppm has no significant influence on pulmonary functions [3]. In fact, the American Conference of Industrial Hygienists (ACGIH) has set a limit to the ammonia concentration in air of 25 ppm in the workplace during a daily working period of 8 hours, and a concentration of only 35 ppm for a short-term exposure time of 15 min [2,3]. Prolonged exposure between 25 ppm to 100 ppm influence the generation of asthma and bronchitis, chronic eye irritation and may cause dermatitis [2]. Concentrations above 100 ppm can produce eye burning, tearing, swollen eyelids, corneal abrasion, blurred vision and even permanent blindness [2–5]. Therefore, the design of novel techniques and sensors that allow the accurate detection of low ammonia concentrations with real time monitoring is quite important [1]. Among the different approaches to detect ammonia it is possible to find those based on the use of Nessler's reagent [6], photoionization detectors [7], semiconductor thin films [8], potentiometric electrodes [9], commercial infrared gas analyzers [10] and sensors based on absorption FET (APSFET) [11]. Although these sensors can detect gaseous ammonia, they exhibit some disadvantages. For instance, Nessler's reagent is a chemical reagent used to detect small amounts of ammonia. However, this reagent is toxic when inhaled, swallowed or absorbed through the skin, and is also a carcinogenic substance. Sensors based on semiconductor thin films exhibit a low selective drift for a particular gas, low reproducibility, weak stability, poor sensitivity and a short sensor active life time. Photoionization detectors exhibit high sensitivity and fast response time, but they need to be calibrated very often to provide accurate measurements. Sensors based on a potentiometric electrodes have the advantage of being sensitive and selective, but they have significant limitations such as relative high power consumption, expensive and requiring the presence of an experienced operator. Regarding sensors operating on APSFET are susceptible to electromagnetic interferences. In the case of commercial infrared gas analyzers they are usually expensive and bulky.

Optical fibers sensors (OFS) are an attractive option due to their inherent characteristics such as good sensitivity, immunity to electromagnetic interference, small size, portability, low cost, and simple light coupling [12,13]. The key concept for employing OFS to detect ammonia is to take advantage of the basicity of ammonia. Therefore, by employing either a pH-dependent dye or -sensitive film which undergoes a suitable fluorescence or color change when exposed to ammonium ions [14–30], ammonia can be measured by tracking absorption changes. The pH-dependent material needs to be attached to the optical fiber and different materials, ranging from sol-gel glasses to polymers, have been used as a trapping matrix. The majority of the sensitive films and dyes are susceptible to variations in temperature and humidity, which can interfere with the detected signal. Therefore, measurements are typically performed in a chamber with controlled ambient conditions.

In this work we demonstrate a fiber optic ammonia sensor based on a universal pH indicator [31]. The universal pH indicator is made of a mixture of different indicators that allows the indicator to exhibit a wide wavelength operating range. Therefore, when the sensor is exposed to ammonia we obtain absorption changes with opposite responses at different wavelengths. This means that the absorption spectrum exhibits a peak whose value is increased, and a valley whose value decreases, as the ammonia concentration is increased. Such behavior is ideal for taking ratiometric measurements that not only enhance the sensor response, but allow us to make the sensor immune to external disturbances such as humidity and temperature. In addition, the indicator is trapped in a commercial aliphatic thermoplastic polyurethane film called Tecoflex^®^ [32]. These films are gas permeable and hydrophobic which means that it is not necessary to soak the sensors in water allowing the operation in drier environments as well as enhance the lifetime of the sensor by preventing the leaking of the indicator. This combination of indicator and film provides a robust and reliable fiber optic ammonia sensor.

## 2. Principle of Operation

**Figure 1.**
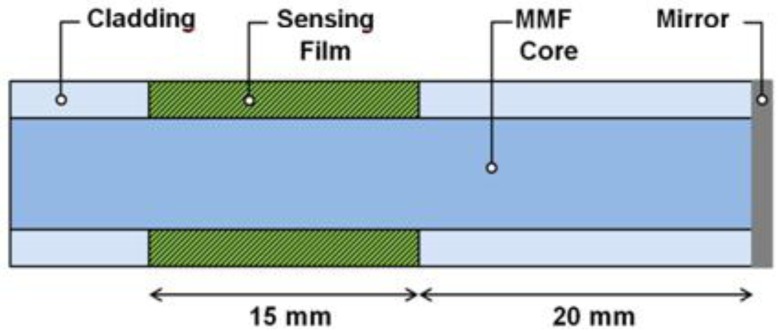
Schematic representation of the sensor head.

As shown in the schematic of Figure 1, the fiber optic ammonia sensor is fabricated using a standard multimode fiber (MMF) structure. The cladding is removed on a small section of the MMF in order to expose the core to the environment. This process allows the evanescent field of the propagating modes to be susceptible to external changes that occur on the MMF surface. Therefore, absorption changes that occur on the MMF surface will be transferred to the spectrum transmitted by the MMF. The universal pH indicator, embedded in a suitable host, is then applied to form a thin film around this un-cladded region. A mirror is also incorporated on the tip of the fiber to reflect the optical signal which also increases the interaction length of the light propagating through the MMF with the pH indicator.

Our sensor is based on a pH universal indicator embedded in an aliphatic thermoplastic polyurethane film, Tecoflex^®^ [32]. When the fiber sensor is exposed to ammonia the universal pH indicator undergoes a color change due to the deprotonation of the indicator which is directly related to the ammonia concentration [25]. The chemical reaction enabling such color changes are explained as follow. The first step is the reaction of gaseous ammonia with water vapor to give ammonium hydroxide, Equation (1):

(1) $${\text{NH}}_{3}(\text{g})+{\;\text{H}}_{2}\text{O}↔{\text{NH}}_{4}^{+}{\text{OH}}^{-}$$

The second step is the indicator deprotonization due to ammonium hydroxide to give a modified form of the universal indicator and water, Equation (2), which causes the color change of the universal indicator:

(2) $${\text{NH}}_{4}^{+}{\text{OH}}^{-}+{\;\text{H}}^{+}{\text{UI}}^{-}↔{\text{NH}}_{4}^{+}{\text{UI}}^{-}+{\text{H}}_{2}\text{O}$$

Finally, the reverse reaction occurs resulting in the initial shape of the universal indicator and ammonia, Equation (3), from the indicator again taking its initial original color:

(3) $${\text{NH}}_{4}^{+}{\text{UI}}^{-}↔{\text{H}}^{+}{\text{UI}}^{-}+{\text{NH}}_{3}(\text{g})$$

Since the color changes and detection mechanism rely on pH variations, these could be also produced by other compounds that undergo similar reactions, such as CO_2_ or NO_2_. However, the cross-sensitivity of the device to different indicators has not been addressed in this work.

It is important to highlight that such color changes are fully reversible, which allows real time monitoring of ammonia concentrations. Color change information is transferred through the MMF via interaction of the evanescent film with the universal pH indicator. The advantage of the universal pH indicator is that it not only exhibits a wide spectral response, but also that the absorption changes in the spectrum have opposite responses. This means that the absorption spectra exhibit a peak and a valley that increase and decrease respectively as a function of the ammonia concentration. Two key advantages can be obtained from this characteristic response. Firstly, we can perform ratiometric measurements (difference between peak and valley absorbance values) that significantly enhance the sensor response to ammonia. Secondly, and more important, the ratiometric measurements allow us to neglect external disturbances such as temperature and humidity. It is well known that pH indicators are sensitive to temperature and humidity, which also occurs in the case of the universal pH indicator. However, when exposed to temperature and humidity changes, both the peak and the valley experience similar changes in absorption, both increase or decrease simultaneously. Therefore, when we measure ammonia under the presence of fluctuations in temperature and humidity, the ratiometric measurement allows us to obtain a correction factor that can eliminate such fluctuations due to temperature and humidity. In addition, the Tecoflex^®^ films are hydrophobic and gas permeable which provides additional advantages to the sensor, such as operation even in extremely dry environments, efficient transport of the element to be measured to the sensitive area of the sensor, and prevent leakage or detachment of the indicator. The combination of the universal pH indicator and Tecoflex^®^ film onto the optical fiber tip produces a sensitive and robust fiber optic ammonia sensor.

## 3. Sensor Fabrication and Experimental Setup

### 3.1. pH Sensitive Solution

The universal pH indicator is provided by the company PANREAC, and is made of a mixture of various indicators such as methyl red (40 mg), *p*-dimethylaminoazobenzene (60 mg), bromothymol blue (80 mg), thymol blue (100 mg) and phenolphthalein (20 mg) [31]. As mentioned before, the advantage of this indicator is that it provides a wide operation range, *i.e.*, absorption changes over a broad wavelength range. In order to immobilize the indicator on the surface of the fiber the polymer is incorporated into a thermoplastic host that allow simple coating of the optical fiber. The pH sensitive solution was prepared by mixing 120 mL of ethanol, 120 mL of pH universal indicator, and 4.32 g of thermoplastic polyurethane (TPU), Tecoflex^®^ provided by the company Thermedics (Newton, NJ, USA). The sensitive film solution was stirred for 180 min at 100 °C in a sealed container before starting the coating process.

### 3.2. Sensor Head Fabrication

**Figure 2.**
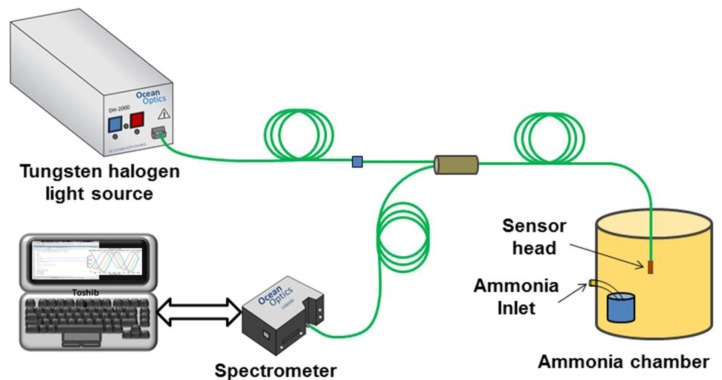
Experimental setup for testing the ammonia sensor.

The sensor was fabricated using the fiber FT-200-EMT from Thorlabs Inc. (Newton, NJ, USA), which is a multimode fiber (MMF) with core and cladding diameters of 200 and 225 μm respectively. The advantage of this MMF is that the cladding is made of polymer which facilitates the removal of the cladding in a specific section of the MMF. The sensor head (Figure 1) is fabricated by first removing the outer protective plastic jacket of the fiber. The MMF was cleaved and the facet is silver coated using a sputtering system (K675XD Quorum Technologies Ltd., (Sacramento, CA, USA). After this, a segment of 1.5 cm of the MMF cladding was carefully removed and cleaned with acetone to remove any remaining polymer waste. This section is located 2 cm away from the tip of the MMF. Finally, the un-cladded region was coated with the pH sensitive solution using a standard dip-coating technique and fully automated deposition system from Nadetech, Inc.^®^ (Pamplona, Spain) The fiber was inserted and pulled out of the solution at a rate of 150 mm/min while the temperature was maintained at 100 °C during the whole process. The coated MMF sensor head was kept at room temperature during 20 min, and then placed into an oven for thermally curing at 85 °C for 15 min. The sensing head was always kept at room temperature for a day before any measurement. The reflection configuration setup (see Figure 2) with the silver coated end faced reduces the size of the sensor and improves the performance of the sensor because the incident light passes twice through the sensitive region.

### 3.3. Experimental Setup

The sensors were characterized using the experimental setup shown in Figure 2. The optical source is a DH-2000-S tungsten halogen light source (Spectral Products, Putnam, CT, USA), with an emission spectrum covering a wavelength range of 215–2,000 nm. The optical source is connected to a bifurcated assembly, UV-200-2 from Ocean Optics^®^ (Ocean Optics, Dunedin, FL, USA), and then sent to the sensing head. The reflected signal from the sensor is monitored using a USB-2000 fiber optic Spectrometer, provided by Ocean Optics^®^. The response of the sensors was characterized using a sealed chamber of 300 mL in order to create a controlled gaseous ammonia environment.

We used aqueous solution of ammonia (1 mol/L density of 0.73 g/L) to obtain concentrations of 10, 25, 50, 50, 75 and 100 ppm into chamber as shown in Figure 2. These concentrations were used to evaluate the performance of the sensor within the permissible levels described before. The sensing head was inserted in the chamber without any contact with the aqueous solution of ammonia.

## 4. Experimental Results

**Figure 3.**
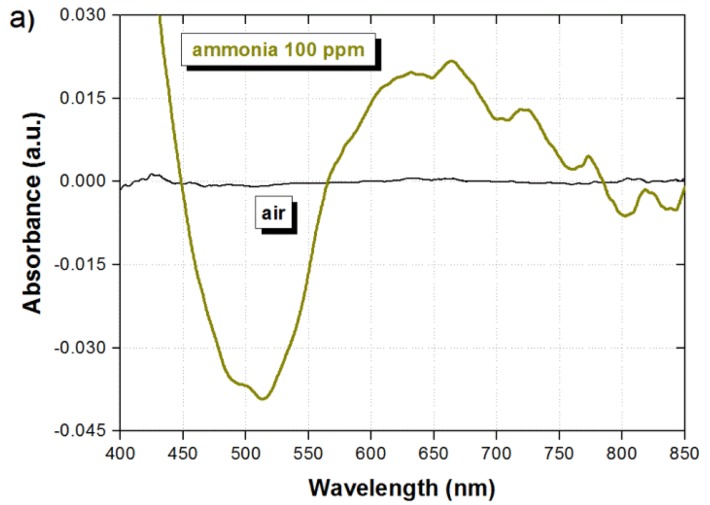

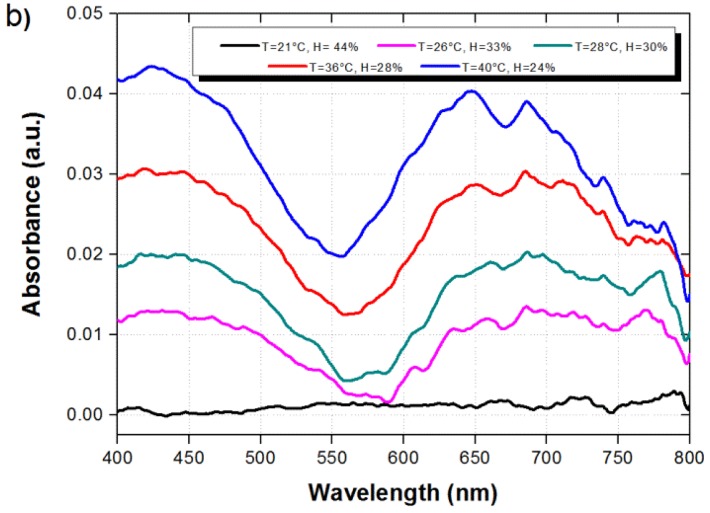
(**a**) Spectral response of the sensor subjected to air and 100 ppm of ammonia, and (**b**) Spectral response of the sensor for different values of temperature and humidity.

The sensor was first characterized to determine its spectral response under the presence of 100 ppm ammonia. Prior to filling the chamber with ammonia, a background reference is recorded by taking the response of the sensor when exposed to air. This background is removed from any further measurement in order to observe the spectral changes resulting only from changes in the chamber atmosphere. As a result, a flat response should be obtained when the sensor is again exposed to air, which confirms that the sensor performance has not been degraded. This is shown by the black line in Figure 3.

The spectral response of the sensor in the presence of 100 ppm of ammonia, after an exposure time of 20 min, is shown by the green line in Figure 3. It is possible to observe two suitable measurement wavelength ranges in Figure 3, where the sensor exhibits maximum absorption changes. A valley is formed around 500 nm as a result of the absorbance reduction, while a peak is formed around 650 nm as a result of the absorbance increase. Such differential response of the sensor is very convenient because, as it has been described earlier and will be shown in the next paragraphs, it enable to perform ratiometric measurements as well as remove external disturbances not related to ammonia. The feasibility of eliminating fluctuations in the sensor response due to external disturbances is better observed by exposing the sensor to different values of temperature and humidity. As shown in Figure 3b, when the temperature and humidity are modified, the absorbance is increased around the wavelengths of 500 nm and 600 nm. In this case, the background was taken at a temperature of 21 °C and humidity of 44%. Since the sensor exhibits opposite response around the wavelength range of 500 nm when ammonia is detected, as compared to temperature and humidity changes, thus by taking the difference between the signals around 500 nm and 600 nm (ratiometric signal) we can eliminate such disturbances due to temperature and humidity while detecting the presence of ammonia. Although not critical under normal operation conditions, temperature and partial pressure, commonly associated to gas adsorption, should be also taking into account because they will also play an important role in the sensitivity of the device.

**Figure 4.**
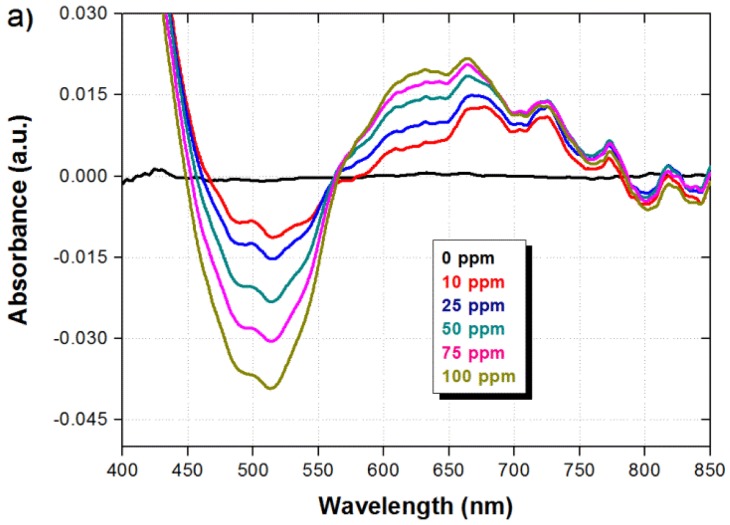

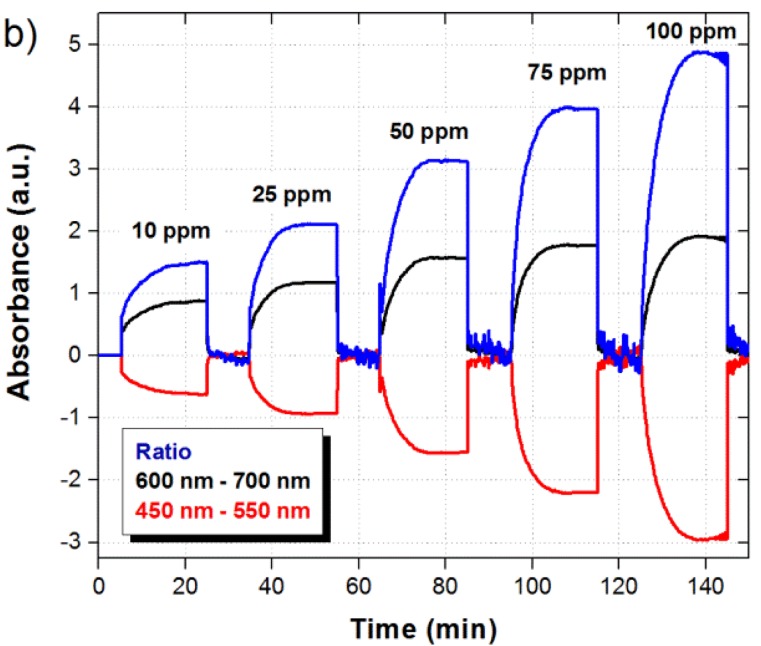
(**a**) Spectral and (**b**) dynamic response of the optical fiber sensor subjected to different ammonia concentrations where ratio is the difference between both signals.

In order to evaluate both, the sensor response in the presence of different ammonia concentrations and the recovery time when ammonia is removed, the sensor was operated in several consecutive cycles within the absence and presence of ammonia. The ammonia concentration was modified from 0 to 100 ppm and the sensor was exposed to ammonia vapor during 20 min in each case. A recovery time in the absence of ammonia (air atmosphere) of 10 min was also allowed between each ammonia concentration. The spectral response of the sensor under the different ammonia concentrations is shown in Figure 4a. It can be observed that the spectral response is modified in a similar way for all concentrations, and only the absorption is decreased/increased around the wavelengths of 500/650 nm as the ammonia concentration is increased. The absorbance variation as a function of the pH/ammonia concentration can be attributed to the utilization of different pH dyes that undergo color changes at different pH/ammonia concentration values. The spectral response from Figure 4a is then integrated within the spectral ranges of 450–550 nm and 600–700 nm, and the difference between both signals is taken to obtain a ratiometric signal (ratio). The dynamic response of the three signals is shown in Figure 4b. As explained before, we can observe that the integrated signals also show opposite response, and the ratio of the signals provide an enhanced and robust response with a good dynamic range. We can also observe that the absorption changes due to the presence of ammonia are fully reversible and the signal returns to its original value after the ammonia is removed. As shown in Figure 4b, the sensor exhibits a response time of five min for the measured value to rise at 80% of the maximum achievable value for each measured concentration. The fall time in all cases is quite fast with a maximum average duration of 18 s.

**Figure 5.**
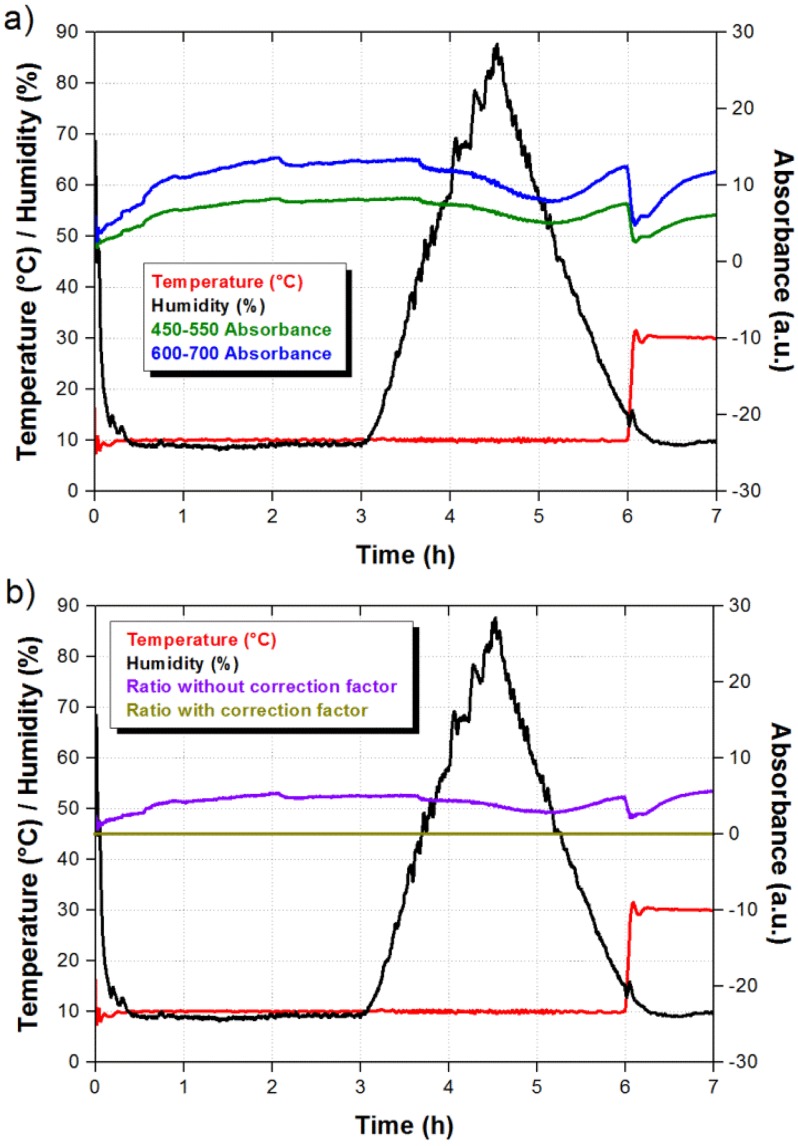
(**a**) Integrated signals as a function of time during humidity and temperature variations; (**b**) Ratiometric signal with and without correction factor

In addition to an enhanced response, the ratiometric signal also allows us to discriminate signal fluctuations due to external factors. It is well known that pH indicators are sensitive to humidity and temperature, and this should also occur in our pH universal indicator. The sensor response to humidity and temperature was evaluated using a climatic chamber model Challenge 250 from Angelantoni Industry (Angelantoni Industrie, Massa Martana, Italy). As shown in Figure 5a, the sensor was exposed to variations from 10% to 85% relative humidity (RH) and a temperature increment from 10 °C to 30 °C which falls outside the humidity changes. The optical absorbance as a function of time was measured at the selected wavelengths ranges and the results are also shown in Figure 5a. We can observe that the sensor response is slow and the absorbance is reduced as the humidity is increased. In the case of the temperature increment we notice an instantaneous reduction of the absorbance, which slowly recovers the absorbance value at the beginning of the temperature increment. However, the most important result is that both integrated signals change not only in the same direction but also with similar proportion. Therefore, when we take the ratiometric measurement we obtain a correction factor that can be applied to the sensor response and eliminate the contributions due to humidity and temperature. As shown in Figure 5b, when the correction factor is not applied the ratiometric signal exhibits small variations as a result from humidity and temperature changes. Nevertheless, a flat response is obtained when the correction factor is taken into account. Since both integrated signals move to opposite directions in the presence of ammonia, the corrected ratiometric signal should exhibit a clear step during the exposure to ammonia.

**Figure 6.**
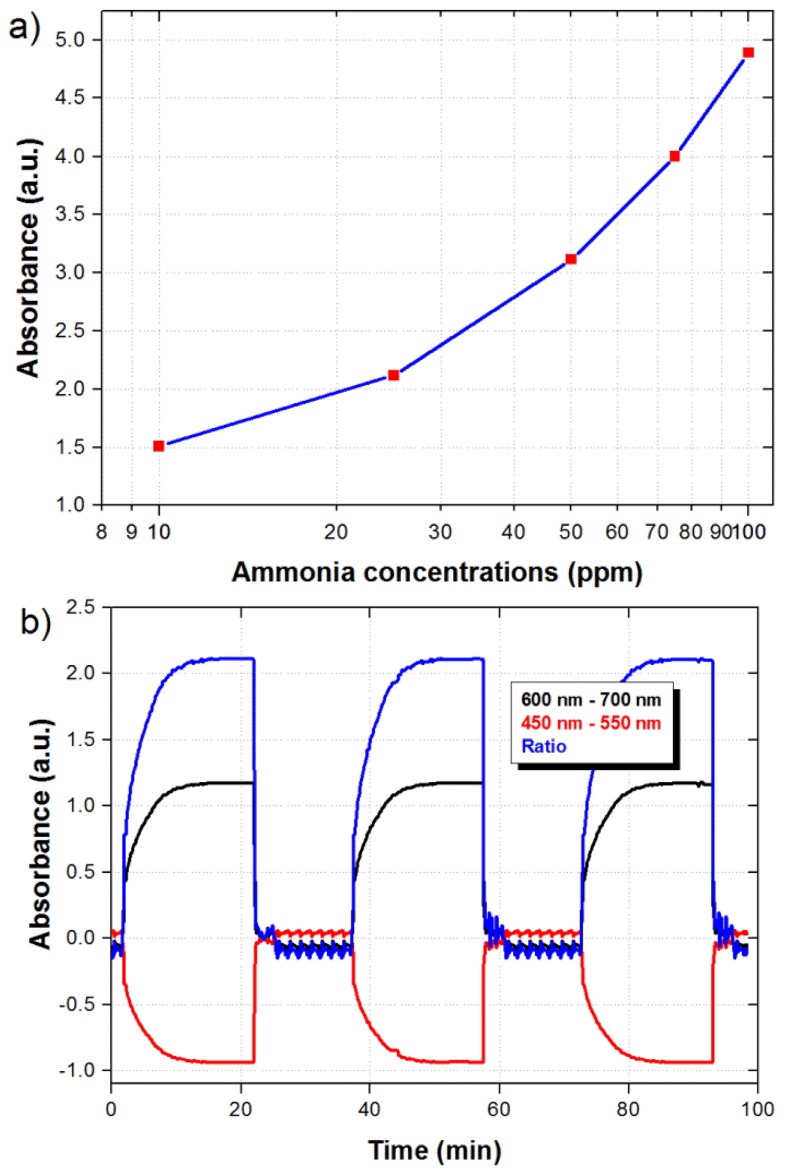
(**a**) Maximum ratiometric signal as a function of ammonia concentration; (**b**) Dynamic response of the sensor when submitted to consecutive cycles at 25 ppm of ammonia.

The maximum ratiometric signal as a function of ammonia concentration is shown in Figure 6a. Although the response is not linear, which is typical in this kind of sensors, the response exhibits an *R^2^* value of 0.9963. The repeatability of the sensor was also evaluated by submitting the sensor to consecutive cycles of 20 min in the presence of 25 ppm of ammonia and 15 min in air. As shown in Figure 6b, the signal reaches the same maximum value every time that the sensor is exposed to the 25 ppm of ammonia.

We can also notice that the fast recovery is preserved after different ammonia measurements. The results demonstrate a highly repetitive behavior of the sensor which is convenient for long term of operation. We should also highlight that the minimum ammonia concentration of 10 ppm detected by the sensor is well below the workplace limit of 25 ppm. Based on the experimental dynamic range we believe the sensor should be capable of detecting smaller ammonia concentrations. Finally, considering the sensor configuration, we have in general three parameters that could be modified in order to improve the sensitivity: film thickness, interaction length, and MMF diameter. Ideally the film thickness could be increased in order to enhance the sensitivity, as long as the optical field interacts with the film. However the sensor response becomes slower since the gas has to penetrate a thicker film. Therefore, a thinner film with a longer interaction length is preferred in this case. We could also reduce the diameter of the MMF in order to allow stronger interactions of the optical mode with the film. Such improvements should also increase the limit of detection of the sensor and are the subject of future work.

## 5. Conclusions

We have fabricated a sensitive fiber optic ammonia sensor using a mixture of pH indicators. The broad wavelength response of the indicator provides a differential response that allows us to obtain ratiometric measurements of ammonia concentrations. The ratiometric measurements provide not only an enhanced output signal, but they also eliminate any external disturbances due to humidity or temperature fluctuations. The indicator was also embedded in a Tecoflex^®^ film whose characteristics enable efficient operation of the sensor under different humidity conditions.

## Notes

**Authors Contributions:** The presented work is a result of the intellectual contribution of the whole team. All members have contributed in various degrees to the development, integration, and test of the system, from the research concept to the experimental design and test results. In particular Adolfo J. Rodríguez fabricated and tested the device and drafted the first version of the manuscript. Francisco J. Arregui and Rene F. Domínguez provided useful suggestions and ideas. Carlos R. Zamarreño and Ignacio R. Matías helped to analyze the characterization results and reviewed the final version of the manuscript and Daniel A. May-Arrioja helped with the simulation results and fully reviewed the manuscript until its final version. All authors read and approved the final manuscript.
